# Effect of Anti‐IL‐23 and Anti‐IL‐17 Therapy on Darier Disease: A Case Series

**DOI:** 10.1111/ajd.70155

**Published:** 2026-06-09

**Authors:** Maione Vincenzo, Zanghi Silvia, Catapano Cecilia, Bighetti Stefano, Cattaneo Isacco, Venturini Marina, Bettolini Luca

**Affiliations:** ^1^ Dermatology Department Spedali Civili, Università di Brescia Brescia Italy

**Keywords:** Darier disease, risankizumab, secukinumab

## Abstract

Darier disease is a rare ATP2A2‐related genodermatosis for which therapeutic options remain limited, as acitretin is often constrained by toxicity and teratogenicity. In this retrospective real‐world case series of eight patients, anti‐IL‐17 therapy showed a more consistent early signal of clinical benefit than anti‐IL‐23 therapy, with greater improvement in disease severity and patient‐reported outcomes and no reported adverse events. Although limited by the small sample size and attrition over time, these findings support further prospective evaluation of biologic therapies in Darier disease.

## 1 Introduction

Darier disease (DD) is a rare inherited genodermatosis caused by pathogenic variants in ATP2A2, which encodes the sarco/endoplasmic reticulum Ca^2+^‐ATPase 2. It is a chronic, relapsing disorder that often markedly impairs quality of life [1]. Among systemic therapies, acitretin is the most consistently reported option for disease control; however, its long‐term use is often limited by cumulative toxicity, including xerosis, alopecia, liver function abnormalities and marked teratogenicity [2].

Recent evidence suggests that inflammation may contribute to DD pathogenesis. In particular, dysregulation of the IL‐17 and IL‐23 pathways has emerged as a possible driver of disease activity, prompting interest in the off‐label use of biologics approved for psoriasis.

We therefore conducted a retrospective real‐world study to describe the outcomes of patients with DD who developed adverse effects during acitretin therapy or showed partial or complete lack of efficacy and were subsequently treated with anti‐IL‐23 and anti‐IL‐17 biologic therapy.

## 2 Main Text

This retrospective cohort study was conducted at the Dermatology Unit of a referral centre in Northern Italy, affiliated with the European Reference Network for rare skin diseases.

All included patients had a confirmed diagnosis of DD established by histopathology. Clinical data were retrospectively collected from routine visits without modification of standard care. All patients had previously provided written informed consent for off‐label treatment and for anonymised use of their clinical data. The study was conducted in accordance with the Declaration of Helsinki, Good Clinical Practice and applicable data protection regulations.

Demographic and clinical variables included comorbidities and previous topical or systemic treatments. Patients intolerant to acitretin, those with acitretin‐related adverse events or those with inadequate response were initially treated with anti‐IL‐23 therapy. In cases of insufficient response or secondary loss of efficacy, treatment was subsequently switched to anti‐IL‐17 therapy using standard psoriasis‐approved dosing regimens.

Risankizumab was selected as the IL‐23 inhibitor because its dosing schedule allowed longer intervals between follow‐up visits. Secukinumab was chosen as the IL‐17 inhibitor on the basis of previously published reports suggesting benefit in DD. Clinical evaluations were standardised and performed every 3 months. Non‐response was defined as either absence of improvement after 6 months of treatment or return to, or worsening beyond, baseline severity.

Disease severity and clinical impact were assessed using body surface area (BSA), Physician Global Assessment (PGA) (added as Data S1), Dermatology Life Quality Index (DLQI) and visual analogue scales (VAS) for pruritus and pain. Severity was also evaluated using the DD Area and Severity Index (DASI), a composite score modelled on the Psoriasis Area and Severity Index. Four anatomical regions were assessed, and for each region, erythema, hyperkeratosis/vegetations, erosions/fissures and extent of involvement were scored. Although DASI has not been formally validated, it was adopted to provide a structured and consistent assessment across physicians. Adverse events were recorded for each biologic therapy.

The anonymised database was created in Microsoft Excel and imported into IBM SPSS version 27.1 for analysis. Continuous variables were summarised as median ± range and categorical variables as absolute frequencies and percentages. Given the small sample size, non‐parametric methods were used.

Longitudinal within‐subject comparisons between baseline (T0) and follow‐up visits (T1 and T2) were performed using the paired Wilcoxon signed‐rank test. Time points were defined separately within each treatment period; T0, T1 and T2 denote sequential visits from the initiation of each biologic (anti‐IL‐23 and anti‐IL‐17 therapy, respectively) and are not shared across periods. Cohen's *d*z. was calculated to estimate the magnitude of change. A responder analysis at T2 defined response as a ≥ 50% reduction in DASI from baseline. All tests were two‐sided, with statistical significance set at *p* < 0.05. Given the retrospective design and the inherent rarity of the condition under study, no formal a priori power calculation was performed. Accordingly, the findings should be interpreted as hypothesis‐generating, providing a preliminary framework for future prospective investigations.

The cohort included eight patients, three men and five women, with a mean age of 46.3 ± 12.8 years. All had a histologically confirmed diagnosis of DD. Comorbidities were heterogeneous and included autoimmune thyroiditis, hypothyroidism, coeliac disease, asthma, fibromyalgia, grade III obesity, latent tuberculosis infection, melanoma in situ, acoustic neurinoma and acute psychotic episodes.

Before biologic therapy, all patients had been treated with acitretin, generally initiated at 25 mg/day and escalated to 0.5–0.7 mg/kg, in combination with topical corticosteroids and antibiotics. Additional therapies included systemic corticosteroids in 37.5% of patients and doxycycline in 12.5%. Adjunctive topical measures such as sodium hypochlorite baths or chlorhexidine‐based cleansers were also recommended. Among the eight patients exposed to acitretin, treatment was discontinued because of elevated liver enzymes in 37.5%, lack of efficacy in 50% and in one patient because retinoid therapy was declined due to pregnancy planning (Table 1).

**TABLE 1:** Clinical characteristics of the analysed patient cohort.

Variable	*n* = 8
Age (years)	46.3 (12.8)
Disease duration (years)	25.5 (10.2)
Gender, *n* (%)	
Female	5 (62.5)
Male	3 (37.5)
Comorbidities, *n* (%)	
Autoimmune thyroiditis	1 (12.5)
Hypothyroidism	1 (12.5)
Celiac disease	1 (12.5)
Asthma	1 (12.5)
Fibromyalgia	1 (12.5)
Grade III obesity	1 (12.5)
Latent tuberculosis	1 (12.5)
Melanoma in situ (history)	1 (12.5)
Acoustic neurinoma	1 (12.5)
Acute psychotic episode	1 (12.5)
Previous treatment, *n* (%)	
Acitretin	7 (87.5)^a^
Elevated liver enzymes	3
Inefficacy	4
Systemic corticosteroids	3 (37.5)
Doxycycline	1 (12.5)

^a^ One female patient declined acitretin due to pregnancy planning.

## 3 Anti‐IL‐23 Cohort

All eight patients entered the anti‐IL‐23 cohort and received risankizumab as first biologic therapy. All completed anti‐IL 23 T1, six reached anti‐IL 23 T2 and only three continued beyond T2.

At baseline, median values were DASI 11.75 ± 9.71, PGA 2.88 ± 0.64, DLQI 10.75 ± 6.26, BSA 26.25% ± 17.05%, VAS pruritus 6.75 ± 2.68 and VAS pain 4.75 ± 3.19. No statistically significant differences were observed between anti‐IL‐23‐T0 and anti‐IL‐23‐T1 or between anti‐IL‐23‐T0 and anti‐IL‐23‐T2. Effect size analysis showed only a small effect for DASI and small effects for pruritus and pain, with a moderate effect for DLQI. No meaningful effect emerged for PGA or BSA. At anti‐IL‐23‐T2, none of the evaluable patients achieved a ≥ 50% reduction in DASI. Although some improvement was observed in the few patients who continued treatment beyond anti‐IL‐23‐T2, these findings should be interpreted cautiously because of attrition and possible survivor bias (Table 2).

**TABLE 2:** Objective and subjective outcomes in patients treated with anti‐IL‐23 and anti‐IL‐17 therapies, respectively.

Outcome	Comparison	*N*	Median T0 (range)	Median FU (range)	*p*	Cohen's *d*z
a. Anti‐IL‐23 patients						
DASI	T0–T1	8	7.0 (2–34)	7.5 (3–35)	1.000	0.046
	T0–T2	6	6.5 (2–13)	6.0 (3–35)	1.000	0.067
PGA	T0–T1	8	3.0 (2–3)	3.0 (2–4)	0.750	0.282
	T0–T2	6	3.0 (2–3)	3.0 (2–4)	1.000	0.221
DLQI	T0–T1	8	11.5 (3–18)	12.0 (3–16)	0.969	0.000
	T0–T2	6	12.0 (3–18)	9.0 (2–20)	0.125	−0.867
BSA	T0–T1	8	17.0 (3–62)	18.0 (3–62)	0.750	0.268
	T0–T2	6	16.5 (3–42)	16.5 (5–62)	1.000	0.091
VAS pruritus	T0–T1	8	7.5 (2–9)	7.0 (0–9)	0.531	−0.269
	T0–T2	6	8.0 (2–9)	6.0 (2–9)	0.250	−0.572
VAS pain	T0–T1	8	4.5 (0–9)	5.5 (0–8)	1.000	0.045
	T0–T2	6	4.5 (0–9)	4.5 (0–9)	0.625	−0.408
b. Anti‐IL‐17 patients						
DASI	T0–T1	6	8.5 (3–36)	7.5 (3–36)	0.406	−0.363
	T0–T2	4	8.5 (4–36)	4.0 (4–13)	0.125	−1.389
PGA	T0–T1	6	3.5 (3–4)	3.0 (2–4)	0.500	−0.645
	T0–T2	4	3.0 (3–4)	2.5 (2–4)	1.000	−0.500
DLQI	T0–T1	6	12.0 (6–18)	12.0 (2–17)	0.688	−0.272
	T0–T2	4	9.5 (5–18)	4.5 (2–14)	0.500	−0.660
BSA	T0–T1	6	24.0 (10–64)	27.0 (10–64)	1.000	0.161
	T0–T2	4	19.0 (10–42)	17.5 (10–42)	0.500	−0.500
VAS pruritus	T0–T1	6	7.0 (4–9)	6.0 (0–8)	0.250	−0.677
	T0–T2	4	6.5 (4–9)	4.0 (0–9)	0.250	−0.866
VAS pain	T0–T1	6	4.0 (1–6)	4.0 (0–6)	0.813	−0.161
	T0–T2	4	4.5 (1–6)	1.5 (0–6)	0.500	−0.866

*Note:* During anti‐IL‐23 treatment, effect size estimation revealed negligible effects on objective disease severity measures (DASI, PGA and BSA), whereas a large effect size was observed for DLQI at T2 (Cohen's *d*z. = −0.87), suggesting a possible improvement in quality of life despite the absence of statistical significance; moderate effects were also noted for pruritus reduction. In contrast, anti‐IL‐17 therapy was associated with consistent numerical reductions in DASI and patient‐reported outcomes at T2, suggesting a potential early clinical signal despite the lack of statistical significance.

## 4 Anti‐IL‐17 Cohort

Six patients with insufficient response or loss of response during anti‐IL‐23 treatment were subsequently switched to secukinumab. The median time from risankizumab discontinuation to secukinumab initiation was 36.5 days (mean ± SD: 34.2 ± 31.4; range 0–69 days; *n* = 6). No formal washout period was planned; in most patients, the switch was intended to be immediate. The observed delay reflects the time required to obtain regional authorisation for rare disease treatment, rather than a deliberate therapeutic washout. Five patients completed anti‐IL‐17‐T1, and four reached anti‐IL‐17‐T2. Only two patients reached anti‐IL‐17‐T6, and only one of these patients was treated up to IL‐17‐T8. Data from later timepoints, T6 and T8, were not included in the main comparative analysis.

Baseline values in this subgroup were DASI 13.17 ± 11.09, PGA 3.17 ± 0.75, DLQI 10.67 ± 5.79, BSA 30.67% ± 18.01%, VAS pruritus 6.33 ± 2.80 and VAS pain 3.67 ± 2.07. Although statistical significance was not reached at either IL‐17‐T1 or IL‐17‐T2, a consistent numerical reduction was observed across objective and patient‐reported outcomes. At anti‐IL‐17‐T2, median DASI decreased to 6.50 ± 5.00, DLQI to 6.25 ± 5.44, BSA to 21.75% ± 13.96%, VAS pruritus to 4.25 ± 3.77 and VAS pain to 2.25 ± 2.63. Effect size analysis suggested clinically meaningful improvement, particularly for DASI and pruritus, with moderate benefit also observed for DLQI. At anti‐IL‐17‐T2, one of four evaluable patients (25%) achieved a ≥ 50% reduction in DASI from baseline (Table 2).

Both risankizumab and secukinumab demonstrated a favourable safety profile, with no adverse events documented throughout the follow‐up period. No patient discontinued treatment due to safety concerns. Where observed, drop‐outs were exclusively related to primary non‐response or secondary loss of response.

DD has traditionally been regarded as a disorder driven primarily by keratinocyte dysfunction, yet increasing evidence supports an additional role for immune dysregulation. Attention has focused particularly on the IL‐17/IL‐23 axis. Haghighi Javid et al. [3] highlighted IL‐17A‐related inflammatory patterns across inherited acantholytic disorders and proposed blockade of this pathway as a therapeutic strategy during inflammatory phases. Ettinger et al. [4] subsequently demonstrated upregulation of Th17‐associated cytokines and genes, together with increased Th17‐cell signatures, in lesional skin from patients with DD. These observations provide a biologically plausible rationale for the off‐label use of psoriasis biologics in selected patients.

Although our study was underpowered to detect statistically significant differences, the overall pattern favoured anti‐IL‐17 therapy over anti‐IL‐23 therapy. Patients receiving secukinumab showed greater reductions in disease severity and more consistent improvements in patient‐reported symptoms, particularly pruritus, whereas risankizumab produced only limited changes in objective disease severity, despite some suggestion of improvement in quality of life. In this context, it should be emphasised that changes in clinical scores reported in this study must be interpreted cautiously and regarded solely as exploratory measures of clinical severity. Accordingly, greater emphasis was placed on patient‐centred outcomes, particularly DLQI and symptom VAS scores.

These findings are broadly consistent with the limited literature. Ettinger and colleagues reported improvement in three patients treated with secukinumab or guselkumab, with therapeutic selection guided by lesional cytokine expression. A paediatric case described by Barajas Mendez et al. also improved with combined secukinumab and acitretin [5]. Giorgio et al. later reported encouraging outcomes with brodalumab, with near‐complete clearance in two patients after 6 months, although limited documentation reduces the generalisability of those findings [6].

A recurring issue across reports, also reflected in our experience, is the heterogeneous and sometimes transient nature of response. Some patients improve rapidly, whereas others show little benefit or relapse after an initial response (Figure 1). Longitudinal work by Ettinger et al. [7] suggested that shifting cytokine profiles and compensatory inflammatory mechanisms may contribute to inconsistent treatment durability, which may help explain why biologic responses in DD appear less stable than in psoriasis.

**FIGURE 1:**
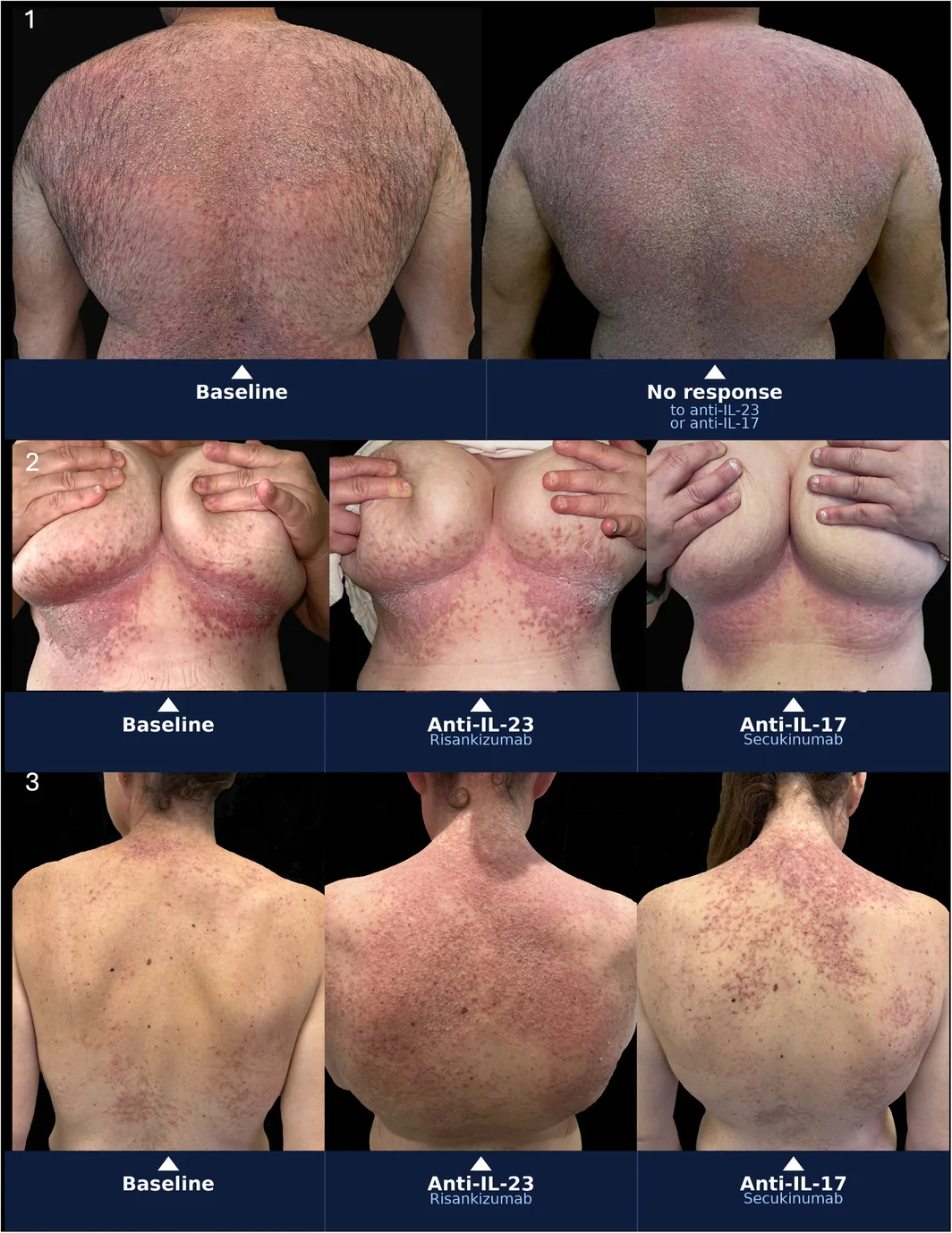
The image shows the different treatment responses of three patients treated with anti‐IL‐17 and anti‐IL‐23 agents. In the first case, the patient showed no changes in the clinical picture. The second case experienced worsening during anti‐IL‐23 treatment, followed by marked improvement with secukinumab. Finally, the third patient showed a clear worsening during treatment with risankizumab and only partial improvement during anti‐IL‐17 therapy, although not compared with the baseline condition.

From a practical standpoint, tissue‐level cytokine profiling may eventually help tailor therapy, but this remains difficult to implement in routine care. A more feasible strategy may be to consider biologics as acitretin‐sparing agents, potentially in combination with low‐dose acitretin, to target both the structural keratinisation defect and the inflammatory component of disease while reducing retinoid‐related toxicity. This approach was not adopted in the present case series, since biologics were introduced only after acitretin had failed or been discontinued because of adverse effects.

Another relevant observation is that even in the absence of a uniform objective response, biologic treatment was frequently associated with improvement in patient‐centred outcomes, particularly pruritus and pain. In a chronic and distressing disorder such as DD, symptomatic relief may be clinically meaningful even without complete lesion clearance.

On the other hand, some authors have suggested that cytokine pathway fluctuations may be directed towards the IL‐13/IL‐4 axis, and that the use of therapies targeting this pathway in patients with DD may improve patient‐reported outcomes [8].

## 5 Conclusion

In conclusion, this real‐world case series suggests that secukinumab may provide greater early clinical benefit than risankizumab in patients with DD who are intolerant of or unresponsive to acitretin. However, selection bias, natural fluctuation of DD, regression to the mean, attrition and potential delayed effects of prior anti‐IL‐23 therapy may all have contributed to the apparent treatment pattern. Therefore, our findings should be regarded as hypothesis‐generating.

Moreover, the small sample size, single‐centre design, attrition over time and lack of validated disease‐specific outcome measures could impact our clinical results.

Nevertheless, together with the emerging literature, our results support further prospective investigation of IL‐17‐ and IL‐23‐targeted therapies in DD, ideally using standardised endpoints and larger future multicentre cohorts, with the aim of improving disease control and quality of life.

## Author Contributions

All authors contributed equally to this work.

## Funding

The authors have nothing to report.

## Consent

The patients in this manuscript have given written informed consent to publication of their case details.

## Conflicts of Interest

The authors declare no conflicts of interest.

## Supporting information


**Data S1:** Treatment pathway—anti‐IL‐23 → anti‐IL‐17 per each patient.

**Table S1:** Physician score assessment for Darier disease.

## Data Availability

The data that support the findings of this study are available from the corresponding author upon reasonable request.
