# Supplementary material for: Effect of Anti‐IL‐23 and Anti‐IL‐17 Therapy on Darier Disease: A Case Series
Source: Australas J Dermatol. 2026 Jun 9;67(7):414–9. doi: 10.1111/ajd.70155 (PMC13741389; doi:10.1111/ajd.70155)
Supplement: Supplementary file 1 [file AJD-67-414-s002.docx]

| 0 : none | No cutaneous lesion |
| --- | --- |
| 1: mild | Sparse and slightly inflammatory papule (that may be slightly hyperkeratotic) |
| 2: Moderate | Numerous hyperkeratotic papules with moderate inflammation(light red color) |
| 3: Severe | Confluent (<30 cm2) hyperkeratotic papules with severe inflammation (brigh red color) |
| 4: Very severe | Confluent and extensive ($\geq$30 cm2) hyperkeratotic papules with very severe inflammation (bright red or purplished color) |

Physician score assessment for Darier Disease
